# Chronic Increase of Urea Leads to Carbamylated Proteins Accumulation in Tissues in a Mouse Model of CKD

**DOI:** 10.1371/journal.pone.0082506

**Published:** 2013-12-04

**Authors:** Christine Pietrement, Laëtitia Gorisse, Stéphane Jaisson, Philippe Gillery

**Affiliations:** 1 Department of Pediatrics (Nephrology unit), University Hospital of Reims, Reims, France; 2 Laboratory of Pediatric Biology and Research, University Hospital of Reims, Reims, France; 3 Laboratory of Biochemistry and Molecular Biology, FRE CNRS/URCA N° 3481, Faculty of Medicine, Reims, France; Universtiy of Maryland Schoool of Medicine, United States of America

## Abstract

Carbamylation is a general process involved in protein molecular ageing due to the nonenzymatic binding of isocyanic acid, mainly generated by urea dissociation, to free amino groups. *In vitro* experiments and clinical studies have suggested the potential involvement of carbamylated proteins (CPs) in chronic kidney disease (CKD) complications like atherosclerosis, but their metabolic fate *in vivo* is still unknown. To address this issue, we evaluated protein carbamylation in the plasma and tissues of control and 75% nephrectomised C57BL/6J mice by LC-MS/MS assay of homocitrulline, the major carbamylation-derived product (CDP). A basal level of carbamylation was evidenced under all conditions, showing that carbamylation is a physiological process of protein modification *in vivo*. CP plasma concentrations increased in nephrectomized *vs.* control mice over the 20 weeks of the experiment (e.g. 335±43 *vs.* 167±19 μmol homocitrulline/mol lysine (p<0.001) 20 weeks after nephrectomy). Simultaneously, CP content increased roughly by two-fold in all tissues throughout the experiment. The progressive accumulation of CPs was specifically noted in long-lived extracellular matrix proteins, especially collagen (e.g. 1264±123 *vs.* 726±99 μmol homocitrulline/mol lysine (p<0.01) in the skin of nephrectomized *vs.* control mice after 20 weeks of evolution). These results show that chronic increase of urea, as seen in CKD, increases the carbamylation rate of plasma and tissue proteins. These results may be considered in the perspective of the deleterious effects of CPs demonstrated *in vitro* and of the correlation evidenced recently between plasma CPs and cardiovascular risk or mortality in CKD patients.

## Introduction

Carbamylation is a nonenzymatic post-translational modification that participates in protein ageing, together with other well-described reactions such as glycoxidation [1] or carbonylation [2]. Owing to the circumstances of the occurence of carbamylation *in vivo*, one could hypothesize that carbamylation is a deleterious process preferentially involved in chronic kidney disease (CKD) and atherosclerosis. CKD independently increases the risk of all types of cardiovascular events [3,4] and induces several other complications consequent to fibrosis [5], insulin resistance [6], erythropoietin resistance [7] and increased suscptibility to infections [8]. The pathogenesis of these complications is complex and still incompletely understood [9]. The identification of new underlying mechanisms and/or risk factors is thus a major goal in order to improve our pathophysiological knowledge of the disease and developing new therapeutic strategies. Among other processes, protein carbamylation has been suggested as a non-conventional risk factor for CKD complications, especially atherosclerosis [10-12]. Carbamylation is characterized by the spontaneous binding of isocyanic acid to free amino groups of amino-acids and proteins, leading to the formation of various carbamylation-derived products (CDPs) [10,11,13]. The most characteristic CDP is homocitrulline, which is derived from the carbamylation of lysine ε-NH2 residues. Isocyanic acid is formed by two major pathways *in vivo*: urea dissociation [13] and myeloperoxidase-mediated catabolism of thiocyanate, principally in atherosclerotic plaques [14,15]. There are also minor environmental sources of isocyanic acid are environmental [16]. Several *in vitro* studies have highlighted the potential involvement of carbamylated proteins (CPs) in degenerative complications linked to CKD [10,11]. This is especially the case with carbamylated lipoproteins, since it has been shown that carbamylated low-density lipoproteins (LDLs) and high density lipoproteins (HDLs) are both dysfunctional and could participate in the progression of the atherosclerotic plaque [17-19]. Additionaly, previous studies by our group have highlighted the deleterious effects of carbamylation on the structural properties of type I and its interactions with inflammatory cells, leading to the stimulation or inhibition of specific functions that could eventually favor infectious and thrombotic complications [20-22].

The pathophysiological hypotheses raised by these *in vitro* findings have been reinforced by the recent publication of the results of three clinical studies (i) demonstrating a relationship between plasma CP concentrations and both mortality in patients undergoing maintenance hemodialysis [23] and adverse outcomes in patients with chronic heart failure [24], and (ii) showing that carbamylated albumin is a potentially modifiable risk factor in CKD patients [25]. 

These series of *in vitro* and *in vivo* results clearly underline the potential involvement of CPs and CDPs in CKD complications. For instance, CP tissue accumulation could be the missing link underlying the increased cardiovascular risk in patients with renal disease. However, no clear data are available on CP formation and accumulation, under either physiological or pathological conditions, and the metabolic fate of these compounds *in vivo* is still largely unknown. So far, only one immunohistochemical study has suggested that CPs are present in the kidney, but the results were not quantitative and the analysis was restricted to one organ [26]. It is critical to understand how CPs are metabolized in order to demonstrate the *in vivo* relevance of the mechanisms demonstrated *in vitro* in order to explain the role of CPs in CKD complications. To obtain this critical evidence, we quantified plasma and tissue homocitrulline in a standard mouse model of subtotal (75%) surgical kidney reduction that mimics CKD, in order to assess the extent of protein carbamylation *in vivo*. We show that carbamylation is a general biological process occurring *in vivo* and that CPs accumulate to a higher extent in tissues with chronic increase of urea, seen in CKD, especially in long-lived extracellular matrix (ECM) proteins such as type I collagen, which could be a source of complications.

## Results

### Plasma concentrations of CPs are higher in CKD mice than in control mice and are correlated with uremia

**Figure 1:**
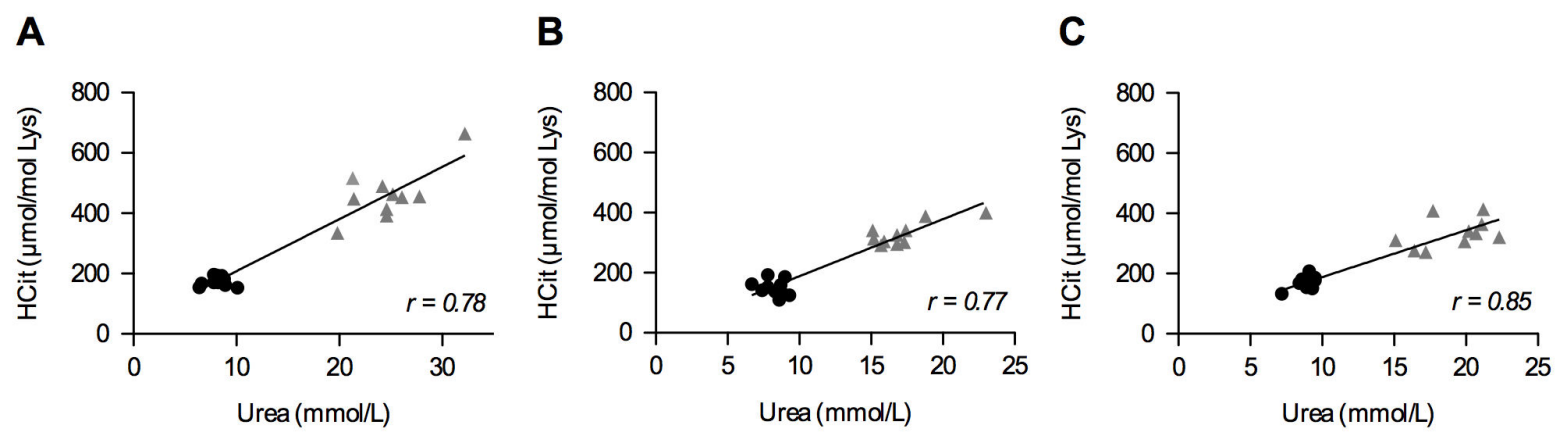
Positive correlation between plasma carbamylated protein and urea. **concentrations after surgery**. Plasma carbamylated protein and urea concentrations were correlated 5 (A), 10 (B) and 20 (C) weeks after surgery. The concentration of carbamylated proteins is expressed as the ratio of homocitrulline (μmol) to lysine (mol). Each dot corresponds to one mouse (10 mice per group). Circles represent sham-operated mice, triangles represent nephrectomised mice. A non-parametric Spearman test was used for the calculation of correlation coefficients, p< 0.0001.

Data on body weight, plasma biochemistry, hematocrit and plasma CPs at baseline and 5, 10 and 20 weeks after subtotal nephrectomy (CKD group) compared to sham operated mice (ShSh group) are shown in Table 1. Subtotal nephrectomy was followed by the appearance of acute renal failure that then progressed to chronic renal failure, as demonstrated by a peak in urea and creatinine levels at 5 weeks followed by a sustained increase in their concentrations 10 and 20 weeks after surgery in CKD mice compared to control mice (urea: at 5 weeks 24.1 mmol/L *vs.* 8.1 mmol/L, at 20 weeks: 18.8 mmol/L *vs.* 9.1 mmol/L; creatinine: at 5 weeks 39.4 µmol/L *vs.* 10.2 µmol/L, at 20 weeks 31.3 µmol/L *vs.* 12.1 µmol/L). Simultaneously, decreases in hematocrit (p < 0.001) and body weight (at 20 weeks: 24.3 g in the shamoperated group *vs.* 22.7 g in the CKD group, p<0.01) were observed. Plasma CP concentrations of homocitrulline expressed relative to lysine (μmol HCit/mol Lys) in the CKD group were 464 + 87 µmol HCit/mol Lys at 5 weeks, 331 + 38 µmol HCit/mol Lys at 10 weeks and 335 + 49 μmol HCit/mol Lys at 20 weeks. CPs were also detected in the plasma of sham-operated control mice but at 2-3-fold lower concentrations: 175 + 16 μmol HCit/mol Lys at 5 weeks, 150 + 26 μmol HCit/mol Lys at 10 weeks and 168 + 22 μmol HCit/mol Lys at 20 weeks (p < 0.001 at all three time points). Urea and plasma CP concentrations were positively correlated at 5, 10 and 20 weeks, with correlation coefficients respectively of 0.78, 0.77 and 0.85 respectively (p<0.0001) (Figure 1).

**Table 1:** Effect of CKD on body weight, serum biochemistry, hematocrit and plasma carbamylated protein concentration before and at 5, 10 and 20 weeks after surgery.

	Sh0		Sh5		CKD5			Sh10		CKD10					Sh20			CKD20			
Body weight (g)	18.9	± 1.0	20.8	± 1.2	19,9	± 0.7	ns	23.6	± 1.0		21.4	± 1.0		***		24.3	± 1.4	22.7	± 0.7		**
Urea (mmol/L)	9.0	± 0.7	8.1	± 1.1	24.1	± 4.2	***	8.2	± 0.8		16.9	± 2.4		***		9.1	± 0.9	18.7	± 2.7		***
Creatinine (µmol/L)	11.2	± 1.9	10.2	± 3.6	39.4	± 11.3	***	5.1	± 2.5		24.3	± 4.7		***		12.1	± 2.9	31.3	± 3.9		***
Calcium (mmol/L)	2.0	± 0.0	1.9	± 0.1	2.4	± 0.2	***	1.8	± 0.1		2.1	± 0.1		***		2.0	± 0.1	2.3	± 0.1		***
Phosphate (mmol/L)	3.2	± 0.4	2.7	± 0.5	2.9	± 0.6	ns	2.1	± 0.3		2.5	± 0.5		ns		2.6	± 0.6	2.9	± 0.3		*
Haematocrit (%)	41.0	± 2.0	42.9	± 1.6	32.3	± 4.8	***	41.2	± 1.7		37.9	± 2.7		**		42.8	± 1.5	35.4	± 1.4		***
Plasma CP (µmol HCit/mol Lys)	186	± 50	174	± 17	463	± 92	***	150	± 27		330	± 34		***		167	± 19	335	± 43		***

Sh : sham operated group, CKD : chronic kidney disease group, CPs : carbamylated proteins. CP concentrations are expressed as ratio of homocitrulline (µmol) to lysine (mol). Values in CKD group are compared to values in Sham group at the same time. Mann-Whitney’s U test: * : p < 0.05 ; ** : p < 0.01 ; *** : p < 0.001 ; ns: not significant.

### CKD increases physiological tissue content of CPs

**Figure 2:**
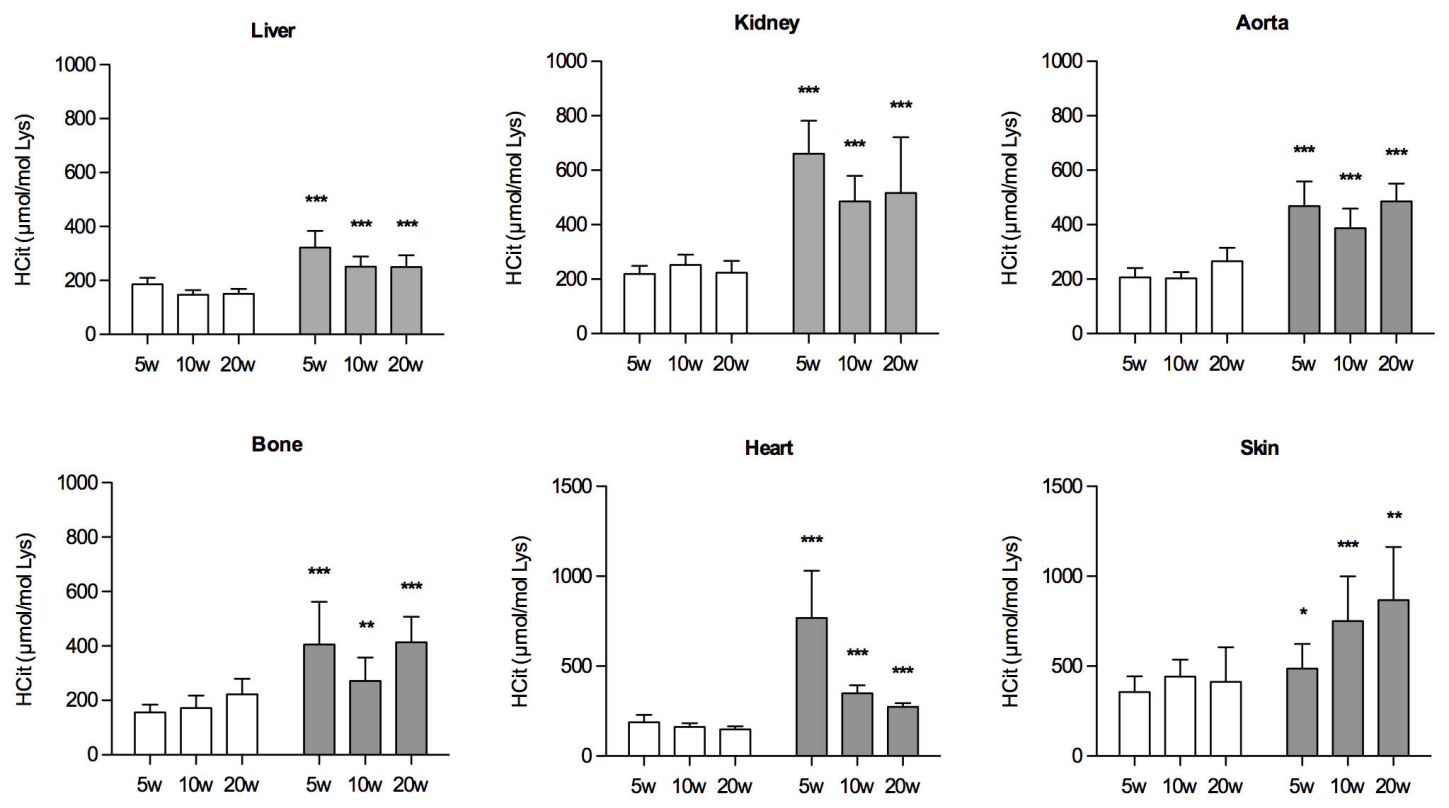
Carbamylated protein content of tissues. The carbamylated protein content of tissues is shown at 5, 10 and 20 weeks after surgery in CKD (grey bars) and sham-operated mice (white bars). Each bar represents the mean value of the 10 mice in the group. CP concentration is expressed as the ratio of homocitrulline (μmol) to lysine (mol). Mann-Whitney U test: *: p < 0.05; **: p < 0.01; ***: p < 0.001.

CPs were detected in all tissues studied (heart, aorta, liver, kidney, bone, skin) in sham operated mice. At baseline, 8-week-old mice had a tissue CP content ranging from 42 to 269 μmol HCit/mol Lys, the maximum value being detected in the aorta (Table 2). This physiological presence of CPs persisted throughout the 20 weeks of the experiment (Figure 2). In CKD mice, the CP contents was 1.4- to 3.9-fold higher than in controls in all tissues studied. The most important differences in CP tissue concentrations 5 weeks after surgery were noted in the heart, kidney and aorta (3.9-, 3.0- and 2.3-fold, respectively) (Figure 2). At 10 and 20 weeks after surgery, CP concentrations were about 2-fold higher in all tissues in CKD mice compared to controls. After 20 weeks, the highest CP content in CKD mice was that of the skin (867 + 296 μmol HCit/mol Lys). A peak in CP levels was observed at 5 weeks in the most vascularized tissues (kidney, heart), but was totally absent in the skin. 

**Table 2:** Tissue carbamylated protein concentrations in 8 weeks-old mice (µmol HCit/mol Lys).

Liver	172	± 24
Kidney	223	± 32
Heart	201	± 36
Aorta	269	± 36
Bone	42	± 15
Skin	216	± 40

**Figure 3:**
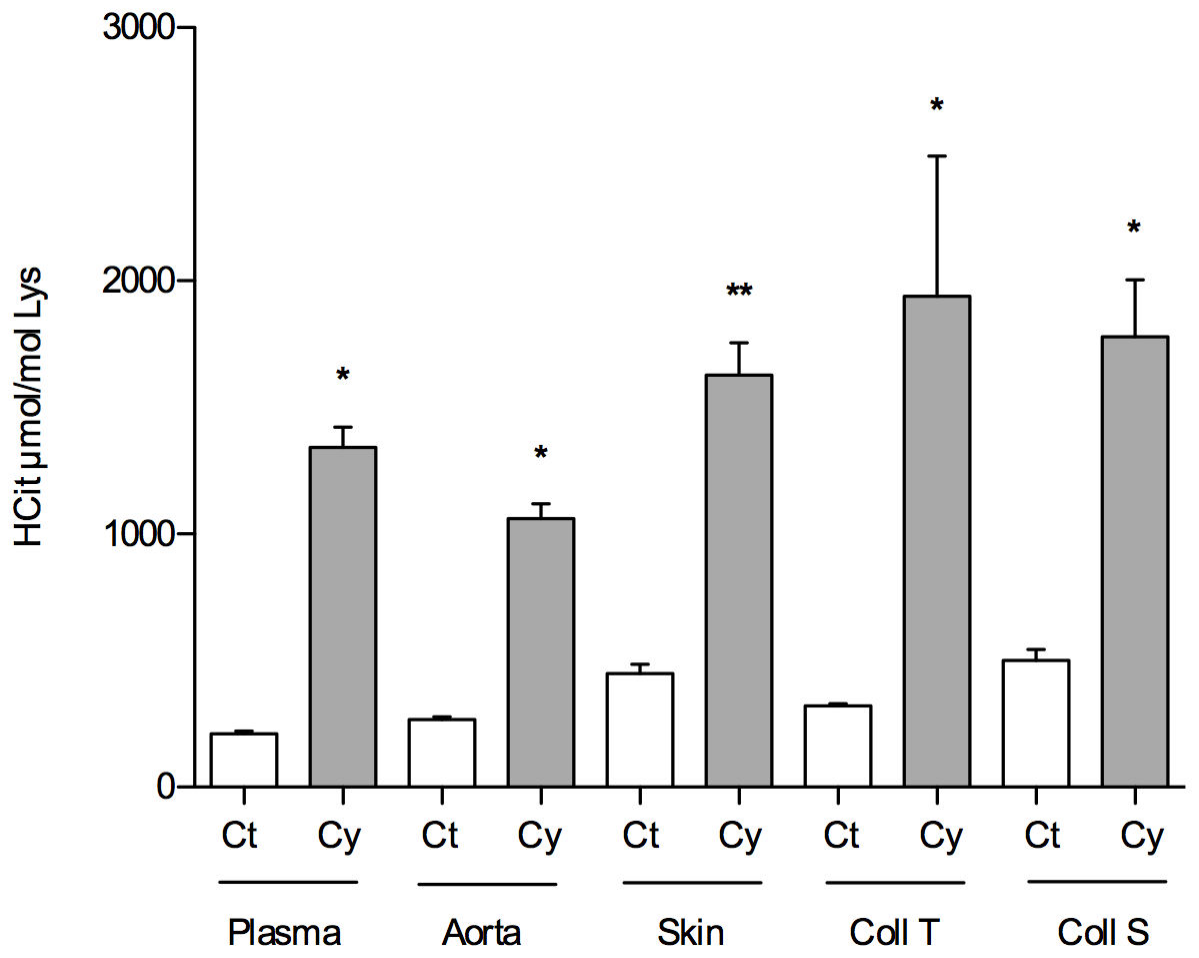
Carbamylation of tissue proteins in the isocyanic acid-consumption model. The carbamylated protein content of the aorta and skin, and purified collagen from the tail (Coll T) and from the skin (Coll S) are shown for mice that received 3 weeks of drinking water containing 0.5 mM sodium cyanate (Cy, grey bars) or normal drinking water (Ct, white bars). Each bar represents the mean value of the 5 mice in the group. CP concentration is expressed as the ratio of homocitrulline (μmol) to lysine (mol). Mann-Whitney U test: *: p<0.05, ** : p<0.01.

Since our aim was to demonstrate the increased tissue carbamylation of proteins secondary to a chronic increase in uremia, we also used a mouse model of isocyanic acid consumption that promotes carbamylation through the increase of plasma of isocyanic acid independently of renal failure. After 3 weeks of isocyanic acid (0.5 mM) addition to the drinking water of mice, there was a significant increase in the concentration of carbamylated proteins in the plasma and tissues analyzed (aorta, skin, purified collagen from skin and tail tendons) (Figure 3): whereas this was no more than 500 μmol HCit/mol Lys in the plasma and tissues of control mice, it was found to be between 1,060 and 1,940 μmol HCit/mol Lys in mice that receiving drinking water supplemented with isocyanic acid.

### The kinetics of CP formation depends on the tissue

**Figure 4:**
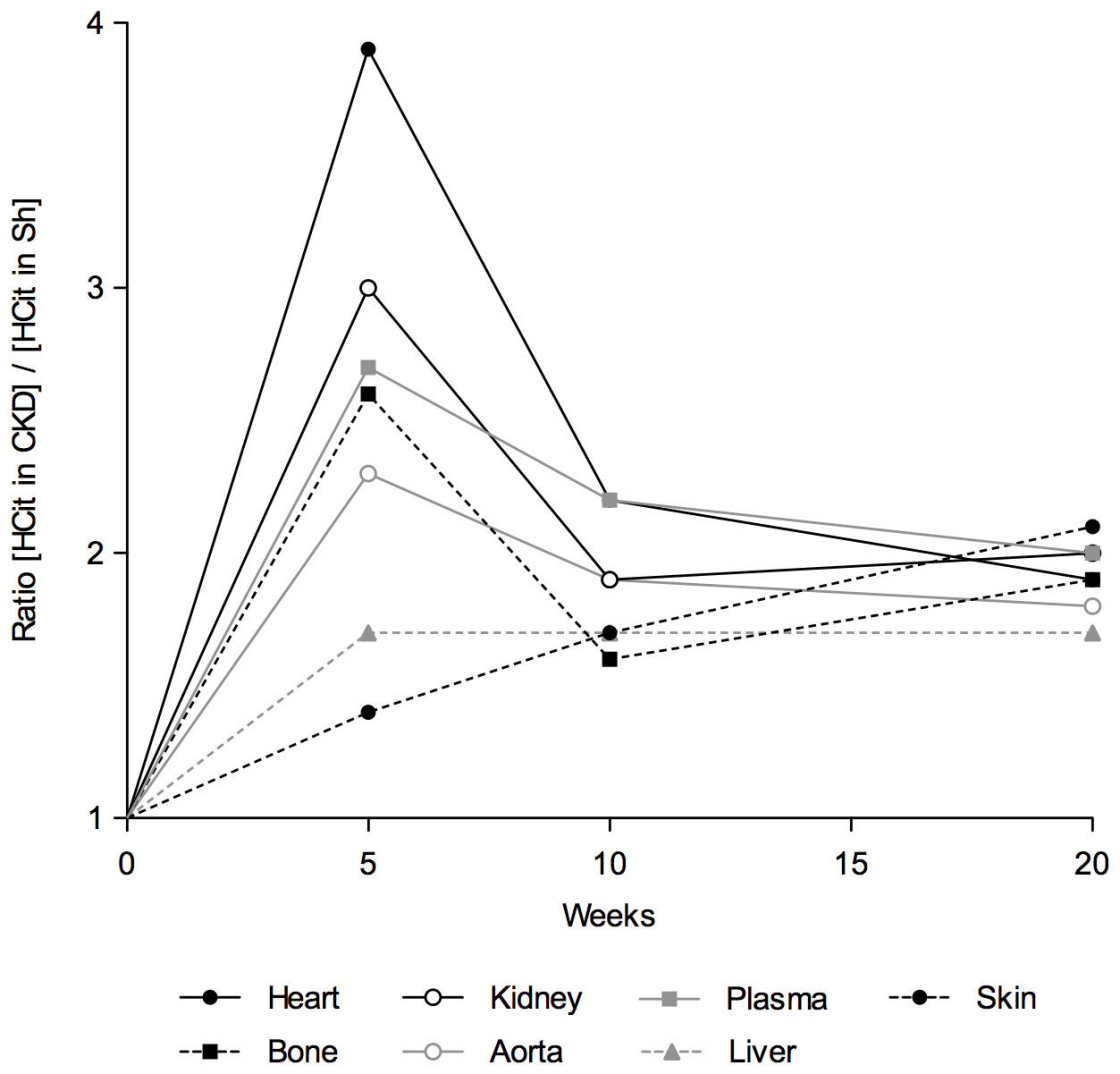
Carbamylated protein accumulation kinetics in plasma and tissues over 20 weeks of CKD. Each point represents the ratio of the mean CP concentration in CKD to that in sham-operated mice (ShSh).

The kinetics of CP accumulation was different depending on the localization studied (Figure 4): The ratios of the plasma CP content of CKD mice to that of the sham group was maximal at 5 weeks (ratio HCit in CKD/ HCit in ShSh: 2.7) but decreased to reach a steady-state level at 20 weeks (ratio: 2.0). The same kind of kinetics, with a maximal difference at 5 weeks and a stable ratio during the following weeks, was also noticed in other tissues, especially in the heart and kidney (heart: 3.9 at 5 weeks, 1.9 at 20 weeks; kidney: 3.0 at 5 weeks, 2.0 at 20 weeks). However, in the skin, the kinetics differed considerably, showing a constant and progressive increase during the 20 weeks of the experiment (ratio: 1.4 at 5 weeks, 1.7 at 10 weeks, 2.1 at 20 weeks). At 20 weeks, the CP content ratio for all the localizations was between 1.7 and 2.1.

### Carbamylated collagen accumulates with time in the ECM during chronic increase of urea, as seen in CKD

**Figure 5:**
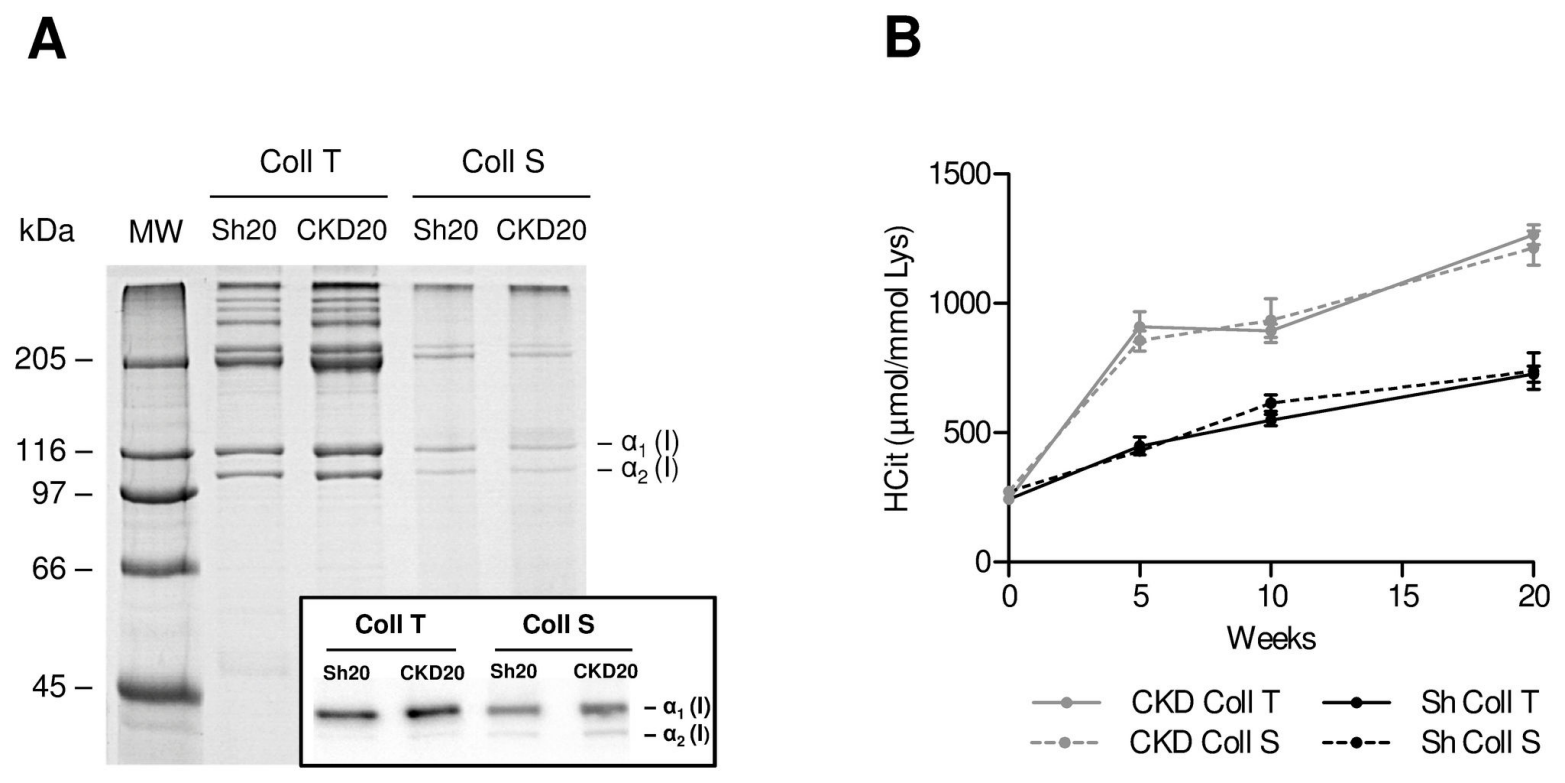
Carbamylation of collagen in CKD mice. (A) SDS-PAGE of collagen extracted from the skin (Coll S) and tail tendons (Coll T). Insert: Western blot of purified collagen. (B) Kinetics of carbamylated collagen accumulation in the tail tendons (CKD Coll T) and skin (CKD Coll S) of the CKD group compared to the sham operated group (ShSh Coll T and ShSh Coll S). Collagen carbamylation was evaluated by homocitrulline quantification, expressed in μmol/mol of lysine. Each dot represents the mean value of the 10 mice in the group.

To strengthen the demonstration of CP accumulation in tissues, we determined the carbamylation rate of the most abundant ECM protein, type I collagen, which should be more prone to carbamylation than other circulating or tissue proteins because of its long biological half-life. For this purpose, collagen was extracted from the skin and tail tendons of sham-operated and CKD mice. Protein integrity was verified by SDS-PAGE which showed α1(I) and α2(I) chains (Figure 5A). The results revealed that carbamylated collagen (CColl) accumulated physiologically over time in the tail and skin of control mice, at the same rate, from baseline (Figure 5C) (8-week-old mice: CColl in skin (CColl S) 271.8 μmol Hcit/mol Lys, CColl in tail (CColl T) 242.3 μmol Hcit/mol Lys) to 20 weeks of experimentation (28- week-old mice: CColl S 737 μmol Hcit/mol Lys, CColl T 726 μmol Hcit/mol Lys). These carbamylation rates remained significantly lower than those observed in CKD mice, althought CColl accumulated at the same rate in the tail and skin of CKD mice as well (Figure 5C). In CKD mice, CColl levels differed significantly from those of control mice starting at 5 weeks after subtotal nephrectomy, and increased throughout the 20 weeks of evolution (at 5 weeks: CColl S 854.7 + 104 and CColl T 907.6 + 183 μmol Hcit/mol Lys *in CKD vs.* CColl S 428.2 + 49 and CColl T 448.5 + 105 μmol Hcit/mol Lys in controls, p< 0.01; at 20 weeks: CColl S 1213 + 208 and CColl T 1264 + 123 μmol Hcit/mol Lys in CKD *vs.* CColl S 737 + 200 and CColl T 726 + 99 μmol Hcit/mol Lys in controls, p< 0.01). This progressive accumulation led to a 5.2- and 4.5-fold increase in CColl T and CColl S respectively between 0 and 20 weeks of CKD.

## Discussion

Patients with CKD have a markedly higher risk of cardiovascular disease and mortality [27-30] which cannot be explained by traditional risks factors alone [29,31]. Carbamylation, which is characterized by the nonenzymatic binding of isocyanic acid to free amino groups of proteins, is a late post-translational modification involved in their molecular ageing. As isocyanic acid is formed by two different pathways *in vivo* (the spontaneous dissociation of urea [13] and the myeloperoxidase-catalyzed metabolism of thiocyanate [15,32]), carbamylation is known to occur in patients with CKD [13,19,33] and to link inflammation and atherosclerosis to form the link between inflammation and atherosclerosis [11,14,15,17]. Many *in vitro* studies have suggested the potential involvement of CPs in degenerative complications linked to CKD [22,26,34,35]. For example, erythropoietin isolated from patients with CKD has a dysfunction associated with increased protein carbamylation [36]. In addition, LDLs isolated from uremic patients and uremic aortic plaques exhibit a higher degree of carbamylation than those from controls, and possess multiple pro-atherogenic properties *in vitro* [17,19,37]. Three recent studies have shown that the quantification of plasma CPs could serve to stratify patients with CKD according to cardiovascular risk and to predict mortality [23-25]. However, neither *in vitro* experiments nor these clinical studies have so far unraveled the pathogenic mechanism linking the increase in plasma CPs with cardiovascular mortality in CKD patients. Therefore, our aim was to quantify tissue CPs *in vivo* during CKD evolution in a mouse model of subtotal (75%) surgical kidney reduction to demonstrate CP accumulation in tissues during CKD and thus provide new arguments in favour of the involvement of carbamylation in CKD complications.

We measured various biological parameters during a 20-week period after kidney reduction in order to verify the establishment of CKD. These measurements indicated that this model of CKD is associated with a first phase of acute renal failure as attested by urea and creatinine peaks observed after 5 weeks. This phenomenon has already been described in other studies using a similar mouse model of CKD [38]. This early urea peak was associated with a peak in plasma CPs suggesting the rapid onset of the carbamylation reaction. Moreover, the kinetics of the formation of plasma CPs was closely dependent on variations in uremia, since CP concentrations decreased between 5 and 10 weeks after surgery to the same extent as uremia. These results indicate that the short half-life of plasma proteins (e.g. 5 days for mouse albumin [39]) prevents the long-term accumulation of CPs in the plasma. They reinforce previous studies from our group that have demonstrated the increase of specific blood CPs during acute renal failure, leading us to propose carbamylated hemoglobin as a marker for this clinical situation [40].

It must be underlined that in sham-operated mice, a basal level of CPs was found in the plasma as well as in all tissues studied, demonstrating that carbamylation may be considered a physiological and ubiquitous process in living organisms, like other nonenzymatic posttranslational modifications such as glycoxidation [41]. The levels of tissue CPs were greatly amplified during CKD, and remained significantly higher than in control mice throughout the 20 weeks following nephrectomy, showing that CPs accumulate more intensely in the organism during CKD. The specific involvement of the carbamylation process in tissue CP accumulation was shown by the increase in the rate of tissue protein carbamylation with the increase in plasma isocyanic acid through the drinking water, a model that mimics high plasma urea levels independently of renal failure [42].

The results of the quantitative evaluation of homocitrulline in our model are in agreement with those of the only previous report showing the presence of CPs *in vivo* by immunohistochemistry, in the kidneys of patients with renal dysfunction [26]. However, the kinetics of CP formation differed according to the tissues studied. As all newly synthesized proteins are over-carbamylated in CKD because of the rapid exposure of their accessible amino groups to elevated isocyanic acid concentrations, their persistence and their accumulation in the organism depend of their turnover. The shorter half-life of plasma proteins may explain the variations of plasma CP concentrations during our experiment (i.e. a peak at 5 weeks and a plateau at 10 and 20 weeks). Accordingly, in highly vascularised tissues such as the heart or kidney, the presence of blood may be reflected in the CP content, so that the CP concentrations of these tissues vary as according to plasma CP content. In contrast, in less vascularized tissues containing a large proportion of long-lived ECM proteins, such as the skin, CPs may accumulate progressively over time. To confirm this hypothesis, we specifically determined the carbamylation rate of a purified ECM protein, type I collagen, which has been demonstrated to be a major target of nonenzymatic posttranslational modifications [41]. Collagen was extracted from the skin and tail tendons and its kinetics showed the progressive accumulation of CColl over the 20 weeks of CKD, which paralleled the progressive accumulation of CPs in skin. It is likely that the observed accumulation of CPs in tissues results from an altered balance between their formation and their degradation. For example, we have previously demonstrated that carbamylation is responsible for the increased sensitivity of type I collagen to proteolysis by gelatinases such as matrix metalloproteinase-2 (MMP-2) and -9 (MMP-9) [21]. Nevertheless, these degradation processes may be insufficient to compensate for the overproduction of new CPs. Our data demonstrating the *in vivo* accumulation of CPs and CColl within tissues during chronic increase of urea, as seen in CKD, may be combined with previous *in vitro* studies to explain the involvement of CPs in the development of long-term complications of CKD, especially those linked to cardiovascular damage and the relentless progression of renal dysfunction. For example, we have previously shown that the carbamylation of only a few lysine residues (i.e. 1 to 11 per α chain) induces subtle local modifications in the triple helical structure of type I collagen impairing its ability to promote fibrillogenesis [22]. CColl networks are more densely packed and made of thinner collagen fibers than native collagen networks [21]. In addition, CColl loses its capacity to stimulate the oxidative function of polymorphonuclear neutrophils [22]. The accumulation of CColl during CKD may contribute to the development of inflammatory syndromes and infectious disorders, which are major causes of morbidity and mortality in uremic patients but are incompletely explained by classic risk factors. This applies especially to CKD-related atherosclerosis. Whereas various studies have suggested that the carbamylation of both LDLs and HDLs alters their functional properties and could promote atherogenesis [17,18], little attention has been devoted to ECM proteins. However, carbamylated ECM proteins like CColl may also play a pivotal role in this process, possibly through their altered interactions with inflammatory cells such as monocytes, which are known to actively participate in the development of atherosclerosis [43]. In particular, CColl promotes monocyte adhesion and strongly stimulates their production of active MMP-9, thus conferring on monocytes an increased ability to degrade the surrounding ECM [20]. The overexpression of MMP-9 increases the risk of rupture of the fibrous cap of the atherosclerotic plaque, and thus represents a key mechanism in thrombotic events such as strokes or acute coronary syndromes. Thus, the presence of CColl may induce an inappropriate remodeling of ECM by activated monocytes, and its accumulation in vessels represents a potential risk factor of cardiovascular events in CKD. This pathway could explain in part the recognition of CKD and atherosclerosis as systemic inflammatory diseases [31,44-46]. Besides, in the vasculature, the carbamylation of ECM proteins may be mediated by myeloperoxidase [15], a granulocyte enzyme known as a mediator of vascular inflammation and plaque vulnerability [32,47-50]. Thus, carbamylation could play a double role in CKD complications: systemic, through urea dissociation, and local, through myeloperoxidase-mediated metabolism of thiocyanate.

To conclude, previous *in vitro* data have established the deleterious effects of carbamylation on protein functions and interactions with cells [11] and suggested the involvement of an increase in CPs as an independent pathogenic factor in atherosclerosis and other characteristic complications of CKD. The results reported here, showing the tissue accumulation of CPs during chronic increase of urea, as seen in CKD, confirm the *in vivo* relevance of these previous findings. They support a pathophysiological explanation for the link between plasma CP concentrations and cardiovascular risk or mortality in CKD.

## Methods

### Chemicals and reagents

Homocitrulline was purchased from MP Biochemicals Europe (Illkirch, France). d8 and d7- citrulline, used as internal standards, were purchased from CDN Isotopes (Quebec, Canada). MS-grade acetonitrile was obtained from VWR International (Strasbourg, France), hydrochloric acid from Merck (Darmstadt, Germany) and other analytical grade reagents from Sigma- Aldrich (Saint-Louis, MO, USA).

### Animals and surgical procedures

All experiments were performed in female C57Bl/6J mice purchased from Charles River (Lyon, France). Animals were fed *ad libitum*, and housed in a room with a constant ambient temperature and a 12-hour light-dark cycle. All animal procedures were conducted in accordance with French government policies (Services Vétérinaires de la Santé et de la Production Animale, Ministère de l’Agriculture) and the study protocol was approved by the institutional animal care committee (Comité d’éthique en expérimentation animale de Reims Champagne Ardenne, registration 56). For the CKD model, sixty 8-week-old female mice were randomly assigned to CKD or sham-operated groups (ShSh). A two-step surgical procedure was used to induce CKD. Surgery was performed under xylazine (Rompun 2%; Bayer, Leverkusen, France) (6 μg/g of body weight) and ketamine (Clorketam 1000; Vetoquinol SA, Lirre, France) (120 μg/g of body weight) anesthesia. The two poles of the left kidney were excised, and one week later the right kidney was removed, reaching a 75% reduction in total renal mass. Control animals underwent sham operations including the decapsulation of both kidneys. At 5, 10 and 20 weeks after surgery, 10 mice from each group (CKD5, CKD10, CKD20, Sh0, Sh5, Sh10, Sh20) were sacrificed under anesthesia (ketamine+xylazyne), and blood was collected in heparinised tubes by cardiac puncture. The heart, liver, kidneys, posterior limb bones, tail, skin and aorta were dissected. Plasma obtained after blood centrifugation and tissues were frozen at -80°C until analysis.

For the isocyanic acid-consumption model, ten 8-weeks-old mice were randomly assigned to two groups (n=5 each) that received normal drinking water (control group, Ct) or drinking water containing 0.5 mM sodium cyanate (Cy group) for 3 weeks, with the renewal of water twice a week. Mice were sacrificed under anesthesia (ketamine+xylazine) and blood and tissues were collected as previously described.

### Blood measurements

Urea, calcium, phosphate and total proteins were measured in plasma obtained by the centrifugation of blood samples, by automated assays using a Modular® analyzer (Roche Diagnostics, Meylan, France). Hematocrit was determined by centrifuging heparinized blood in a microcapillary tube.

### Tissue extraction

Approximately 100 mg of tissues (10 mg of aorta) were homogenized with 1 mL of 0.5 M acetic acid in Lysing Matrix D tubes with the FastPrep-24 apparatus (MP Biochemedicals, Illkirch, France). After homogenization, samples underwent pepsin digestion (10% (w/w)) for 24h at 37°C.

### Collagen purification

Type I collagen was extracted from the skin and tail tendons as previously described24. Briefly, collagen from tail tendons was extracted with 0.5 M acetic acid for 24h at 4°C and precipitated with 0.7 M NaCl. For skin collagen extraction, skin samples were homogenized using FastPrep-24 aparratus in 0.5 M acetic acid containing 1‰ (w/w) pepsin and incubated in this solution for 24h at 4°C, before precipitation with 0.7 M NaCl. In both cases, precipitates were solubilized with 18 mM acetic acid and dialyzed against distilled water for 3 days at 4°C. The samples were then freeze-dried and stored at -80°C until analysis. The integrity of extracted collagens was checked by SDS-PAGE and western blotting. Briefly, proteins were separated by 6% (w/v) SDS-PAGE and transferred to a PVDF membrane. The membrane was blocked with Tris-buffered saline (Tris HCl 20mM pH 7.5, NaCl 150mM) containing 0.1% Tween (v/v) (TBS-T) and 5% (w/v) non fat dry milk, at room temperature for 2 hours. The membrane was incubated overnight at 4°C with an anti-type I collagen antibody (Millipore, Molsheim, France, used at 1/8000 dilution in TBS containing 1% dry milk). After several washes with TBS-T, the membrane was incubated for 1 hour at room temperature with a peroxidase-conjugated anti-rabbit IgG antibody (GE Healthcare, Velizy- Villacoublay, France) diluted 1/10000 in TBS-T). Finally, detection was performed using an ECL detection kit (GE Healthcare) according to the manufacturer’s protocol. The purity of extracted collagens was also determined by amino acid composition, performed by anion exchange chromatography (Hitachi L-8800 analyzer, ScienceTec, France). The amino acid composition showed about 30% glycine residues and 10% proline/hydroxyproline residues, which is consistent with the primary sequence of type I collagen.

### Carbamylated protein quantification

The carbamylation rate of proteins was evaluated by HCit quantification, according to a previously described technique [51], with slight modifications. For this purpose, plasma, tissue extracts and purified collagen were subjected to acid hydrolysis with 6M hydrochloric acid for 18h at 110°C. Hydrolysates were evaporated twice to dryness under a nitrogen stream. Dried samples were resuspended in 100 μL of 125 mM ammonium formate containing 1 μM d7-citrulline, and filtered using Uptidisc PTFE filters (4 mm, 0.45 μm, Interchim, Montluçon, France). Samples were then diluted 10-fold in 125 mM ammonium formate (containing 1 μM d7-citrulline) and subjected to chromatography before LC-MS/MS analysis (API4000, ABSciex, Les Ulis, France). 

Since CKD does not interfere with the amino acid composition of proteins, homocitrulline values were compared to lysine content in the hydrolysates, and expressed as the ratio of homocitrulline to lysine (HCit/Lys). Lysine quantification was performed by LC-MS/MS as follows: the separation was performed on a Kinetex HILIC column (100 x 4.6 mm, 2.6 μm (Phenomenex, Le Pecq, France)) preceded by a guard column packed with the same resin. Mobile phase A consisted of 5 mM ammonium formate (pH 2.9) and mobile phase B was 100% acetonitrile. The flow rate was constant at 0.9 ml/min during all separation steps. The gradient program was as follows: 0-0.5 min: 90% B; 0.5-1 min: gradient to 50% B; 1-2 min: 50% B; 2-2.5 min: gradient to 10% B; 2.5-3.6 min: 10% B; 3.6-3.8 min: gradient to 90% B; 3.8-5.5 min: 90% B. The injection volume was 1 μL and oven temperature was set at 30°C. Before injection, filtered samples were diluted 10-fold in 125 mM ammonium formate buffer and then diluted 20-fold in 5mM ammonium formate buffer pH 2.9; the same two buffers containing 65 μM d8-lysine were used as internal standards. Detection was performed in positive-ion mode and the electrospray ionization (ESI) source parameters were as follows: curtain gas: 40 psi; collision gas (CAD): 6 psi; nebulization gas 1: 40 psi; nebulization gas 2: 60 psi; ion-spray voltage: 4500 V, and source temperature: 650°C. Nitrogen and argon were used as nebulization and collision gases, respectively. The parameters used for mass detection by multiple reaction monitoring (MRM) were as follows: 147.1>84.1, CE: 27 and CXP:16 for lysine and 155.0>92.0, CE:19 and CXP:6 for the internal standard.

### Statistical analysis

The CKD and sham-operated groups were each composed of 10 mice, while the isocyanatefed and control groups were composed of 5 mice. Homocitrulline values were compared between the different groups using the Mann-Whitney U test. Differences were consideredstatistically significant when the p-value was ≤ 0.05. A non-parametric Spearman test was used for the calculation of correlation coefficients.
